# ALLocator: An Interactive Web Platform for the Analysis of Metabolomic LC-ESI-MS Datasets, Enabling Semi-Automated, User-Revised Compound Annotation and Mass Isotopomer Ratio Analysis

**DOI:** 10.1371/journal.pone.0113909

**Published:** 2014-11-26

**Authors:** Nikolas Kessler, Frederik Walter, Marcus Persicke, Stefan P. Albaum, Jörn Kalinowski, Alexander Goesmann, Karsten Niehaus, Tim W. Nattkemper

**Affiliations:** 1 Biodata Mining Group, Bielefeld University, Universitätsstraße 25, 33615 Bielefeld, Germany; 2 Department of Proteome and Metabolome Research, Center for Biotechnology, Bielefeld University, Universitätsstraße 25, 33615 Bielefeld, Germany; 3 Microbial Genomics and Biotechnology, Center for Biotechnology, Bielefeld University, Universitätsstraße 27, 33615 Bielefeld, Germany; 4 Bioinformatics Resource Facility, Center for Biotechnology, Bielefeld University, Universitätsstraße 25, 33615 Bielefeld, Germany; 5 Bioinformatics and Systems Biology, Justus Liebig University Gießen, Heinrich-Buff-Ring 58, 35392 Gießen, Germany; 6 CLIB Graduate Cluster Industrial Biotechnology, Center for Biotechnology, Bielefeld University, Universitätsstraße 27, 33615 Bielefeld, Germany; Swiss Institute of Bioinformatics, Switzerland

## Abstract

Adduct formation, fragmentation events and matrix effects impose special challenges to the identification and quantitation of metabolites in LC-ESI-MS datasets. An important step in compound identification is the deconvolution of mass signals. During this processing step, peaks representing adducts, fragments, and isotopologues of the same analyte are allocated to a distinct group, in order to separate peaks from coeluting compounds. From these peak groups, neutral masses and pseudo spectra are derived and used for metabolite identification via mass decomposition and database matching. Quantitation of metabolites is hampered by matrix effects and nonlinear responses in LC-ESI-MS measurements. A common approach to correct for these effects is the addition of a U-^13^C-labeled internal standard and the calculation of mass isotopomer ratios for each metabolite. Here we present a new web-platform for the analysis of LC-ESI-MS experiments. ALLocator covers the workflow from raw data processing to metabolite identification and mass isotopomer ratio analysis. The integrated processing pipeline for spectra deconvolution “ALLocatorSD” generates pseudo spectra and automatically identifies peaks emerging from the U-^13^C-labeled internal standard. Information from the latter improves mass decomposition and annotation of neutral losses. ALLocator provides an interactive and dynamic interface to explore and enhance the results in depth. Pseudo spectra of identified metabolites can be stored in user- and method-specific reference lists that can be applied on succeeding datasets. The potential of the software is exemplified in an experiment, in which abundance fold-changes of metabolites of the l-arginine biosynthesis in *C. glutamicum* type strain ATCC 13032 and l-arginine producing strain ATCC 21831 are compared. Furthermore, the capability for detection and annotation of uncommon large neutral losses is shown by the identification of (γ-)glutamyl dipeptides in the same strains. ALLocator is available online at: https://allocator.cebitec.uni-bielefeld.de. A login is required, but freely available.

## Introduction

Metabolomics is the systematic analysis of the set of metabolites that are synthesized by an organism – also known as the metabolome [1], [2]. The analysis involves different steps to get from the wet-lab experiment to an evidence or assumption of biological significance. One of the workhorses for the measurement of small molecules in biological samples is liquid chromatography coupled to mass spectrometry (LC-MS), using electrospray ionization (ESI). But it is not the data acquisition that is posing the greatest challenge to metabolomics: In a survey from 2009, asking for the greatest bottleneck of metabolomics, 35% of the respondents named the identification of metabolites the biggest challenge, 22% thought that assigning biological significances is most important, and 14% decided that data processing/reduction is the crucial bottleneck [3].

The identification of truly novel compounds is not possible by mass-spectrometry alone, but requires complementary analytical techniques such as NMR. Metabolite identification in the context of mass-spectrometry based metabolomics rather means assigning possible known molecular entities to all detected peaks or peaks of interest. Using electrospray ionization, peaks can be observed representing so called pseudo-molecular ions. Here, intact analytes build adducts with small inorganic ionic species. Determining m/z-values with high accuracy allows the determination of a reasonable number of possible sum formulae for each adduct by mass decomposition. Previous recognition of the type of adduct ([M+H]^+^, [M+Na]^+^, etc.) supports narrowing down the list of candidates.

Mass spectra created by LC-ESI-MS pose unique challenges on interpretation. During analysis different adducts and fragments of the original metabolite are formed and thus can be found as mass signals. For a proper identification and quantitation of the original metabolite, these signals have to be associated and annotated. In case two given molecules M_1_ and M_2_ could not be separated by retention time in the chromatographic step they have to be separated in artificial spectra that contain only those peaks which originate from the same analyte M_1_ or M_2_, also referred to as pseudo spectra. Such a pseudo spectrum might for example comprise the peaks of the hydrogen ion adduct and the ion fragments created through the losses of water or ammonia ([M+H]^+^, [M+H-H_2_O]^+^ and [M+H-NH_3_]^+^ respectively), which all derive from the same molecule M. Formed ions may even be ambiguous. For example, a mass difference of 17.027 Da can be explained by the neutral loss of ammonia ([M+H-NH_3_]^+^ and [M+H]^+^) or by the formation of an ammonium adduct ([M+H]^+^ and [M+NH_4_]^+^). This is highly dependent on many technical parameters, for example mobile phase composition and ion optic settings.

One of the most powerful data analysis packages for untargeted metabolomic profiling is XCMS [4], [5], providing means for peak detection, retention-time alignment, data annotation, and statistics. To solve the before mentioned problem of mass spectral deconvolution, XCMS interacts with the CAMERA tool [6] that assembles pseudo spectra of peaks with high retention time correlation and identifies isotopes, common adducts and losses. For the annotation of fragment peaks, the tool requires all potential losses to be predefined and thus does not cover more compound specific (uncommon) losses. However, identification of fragment peaks can provide structural information that might help to distinguish between isobaric compounds. XCMS and CAMERA are available through the web platform XCMSOnline [7], which allows conducting and exploring the fully automated processing, but does not provide any possibility to easily curate these results. Another freely available framework for LC-MS data processing, visualization and analysis is MZmine 2 [8], [9]. This software also offers automated peak identification, including the detection of common adducts and matching of calculated neutral masses to chemical databases. Most importantly, peaks representing fragments of analytes in the full scan MS data are detected by matching peaks to multistage MS spectra generated in the same run. However, automatic identification of fragments for one-dimensional MS data is not supported. With MET-COFEA another platform software was published recently [10], combining novel mass trace based extracted-ion chromatogram (EIC) extraction, continuous wavelet transform (CWT)-based peak detection, and compound-associated peak clustering and peak annotation algorithms.

One major aspect of LC-MS based metabolomics that is only recently stepping into the focus of cheminformatics is metabolite quantitation via isotopic labeling. The use of stable isotopic labeling (SIL) has become an important and popular approach in the field of metabolomics. Many strategies using SIL were developed, enabling more accurate metabolite identification and quantitation in complex biological samples [11]–[13]. The numerous advantages of this common approach have been reviewed recently [14]. Common to most SIL experiments is the mixing of naturally labeled (unlabeled) samples with samples that are enriched with stable isotopes and the analysis of these mixed samples by GC- or LC-MS. Either one group of samples from one experimental condition is unlabeled and another set of samples from a second experimental condition is labeled, or both groups of samples are unlabeled and a labeled internal standard is added to each sample. In any case, this allows calculating abundance ratios of metabolites in the two samples, while matrix effects can be neglected [11], [13]. Additionally, the distance between the signals of the unlabeled and fully labeled isotopologue peaks provides substantial benefits for metabolite identification as it can be used to infer the correct number of atoms of the respective element in the analyte. This facilitates a more precise calculation of sum formulae. The software tool mzMatch-ISO [15] offers the necessary preprocessing for that and consequently allows to associate ^13^C peaks to their respective ^12^C counterparts, thus providing the basis to generate ratios. mzMatch-ISO however lacks support to identify the adducts and losses of complex LC-ESI-MS spectra. This holds also true for the commercially available Isotopic Ratio Outlier Analysis (IROA) software (NextGen Metabolomics, Michigan, USA) [16]. In 2012 an algorithm and program (MetExtract) was published that associates monoisotopic unlabeled and monoisotopic labeled peaks of the same metabolites [17]. It uses the mass difference between the two peaks and the charge that is inferred from the isotopic pattern to calculate the corresponding number of atoms of the labeling element (e.g. carbon). Furthermore, it assembles peaks of extracted predefined adduct-, fragment- and polymer ions into peak groups. Only recently, the XCMS package was extended for the analysis of isotopically labeled compounds by the introduction of X^13^CMS [18]. X^13^CMS associates e.g. (U-)^13^C-labeled peak groups to their corresponding unlabeled peak groups in another measurement. This is taken as a basis for differential analyses.

The current landscape of metabolomics software provides solutions for each step of the entire processing from LC-MS raw data, to signal processing, to metabolite identification and relative quantitation. Nevertheless, it misses one that (a) uses the full potential of ^13^C-stable isotopic labeling for metabolite and fragment annotation, (b) is optimized for mass isotopomer ratio analysis, (c) provides users with an interactive interface not only to explore but also to modify the results of automatic processing, and finally (d) addresses the strong and well advanced evolution of research projects towards cross-group collaborations [19]. To fill these gaps we developed the ALLocator system, presented in this manuscript. ALLocator is a novel web-platform particularly for the comprehensive analysis of metabolomics LC-ESI-MS (labeling) experiments and is streamlined for mass isotopomer ratio analysis. It covers all aspects (a) - (d), as shown in our application example.

**Figure 1:**
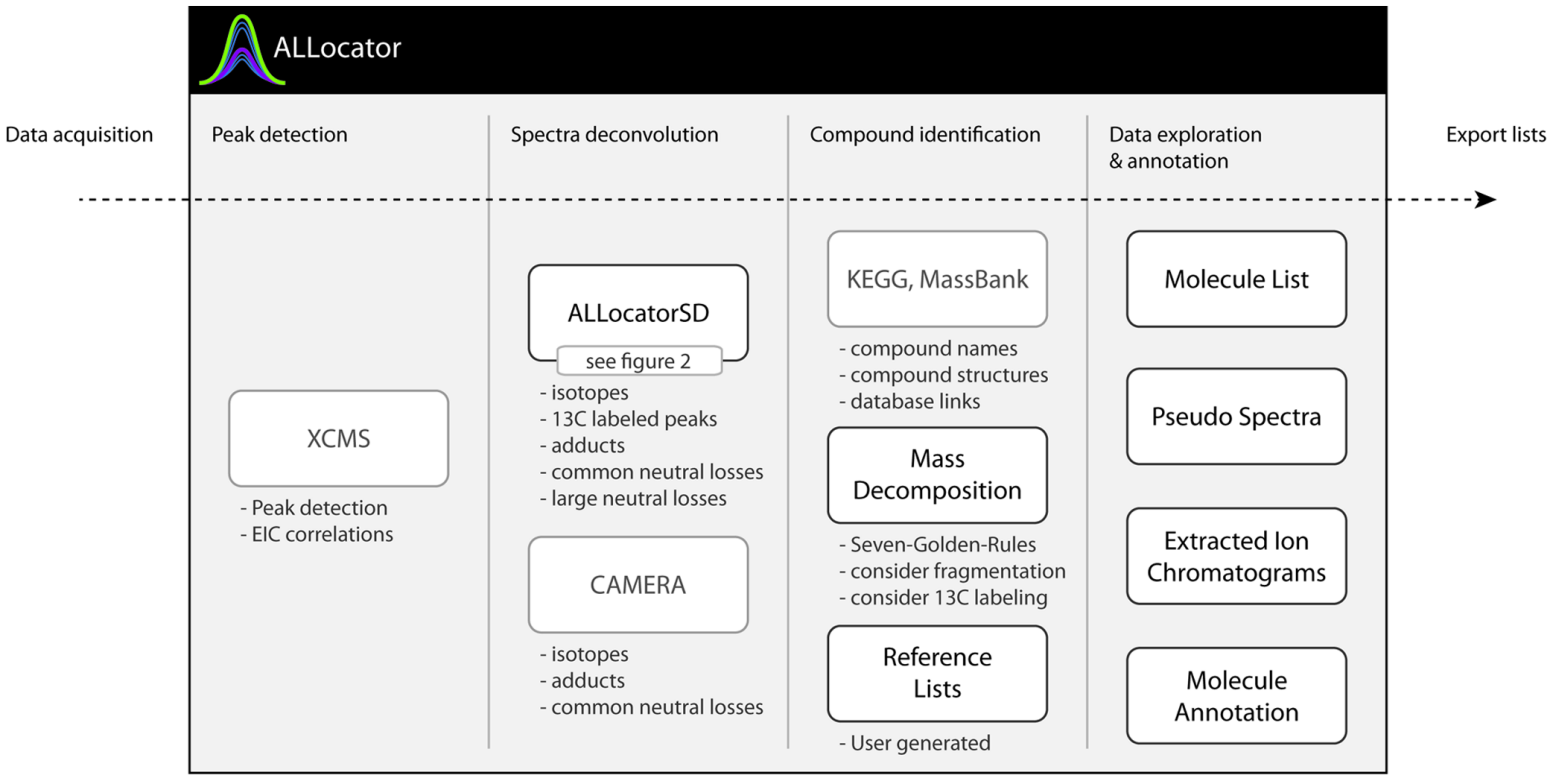
The workflow covered by the ALLocator web platform (integrated third-party features are shown in light gray): After raw data upload, the first step is peak detection. Second, either the new ALLocatorSD algorithm or CAMERA can be applied for spectra deconvolution. Next, tools are provided to identify and annotate detected compounds. Then, data exploration is facilitated through dynamic and interactive visualizations that directly allow to confirm or to modify results of the automated processing steps. In the final step, results can be exported for external use.

ALLocator is an integrative data analysis system, so users can solve as many tasks in one system (with one interface) as possible, in order to generate datasets that can be used for statistical analyses. It covers the entire workflow of data annotation, beginning from uploading raw chromatogram data, to peak detection, to spectra deconvolution, to compound identification, and finally to data exploration and annotation (see Figure 1). The core feature is the new processing pipeline for spectra deconvolution ‘ALLocatorSD’. It is optionally capable of dealing with data derived from ^13^C-labeling experiments, and the use of this information to detect even large uncommon losses. ALLocatorSD will be described in very detail in this manuscript. The results of the pipeline can then be used to identify each small molecule via different (semi-) automated or manual ways. All generated data can be explored and curated with interactive and dynamic visualizations. The compound identification methods, data exploration and manual annotation features can also be applied to results achieved with the CAMERA tool [6], which is integrated into the ALLocator web platform as an alternative approach. To ensure long-term use of manual metabolite annotation efforts, ALLocator provides the possibility to generate and make use of user- and protocol-specific reference databases.

## Implementation and Methods

The ALLocator web platform comprises methods and tools for the semi-automated analysis of LC-ESI-MS experiments, from the import of chromatographic raw data, to the export of lists of annotated and quantified compounds. Users can create experiments and upload chromatograms (CDF-, mzXML-, mzData –files) of both, positive- and negative-mode measurements. The web interface then guides the user through the customizable pre-processing steps, and finally displays the results in interactive and dynamic visualizations for data exploration and manual annotation. The general concept of all features is to achieve transparency of the data, i.e. to provide researchers with all information to support decisions in peak annotations, rather than to plot irrevocable results of black box algorithms.

### Pre-processing: Peak Detection and Spectra Deconvolution

Pre-processing algorithms that are offered by ALLocator can be started either for a single chromatogram or for all the chromatograms of an experiment at once. Users can set parameters for these algorithms through the web interface. These pre-processing “jobs” are submitted to the compute cluster of the Center for Biotechnology of Bielefeld University (CeBiTec), hosted by the Bioinformatics Resource Facility (BRF). Whenever the Java software has to call programs running in the R environment [20] (version 2.13.2), this is realized through the Runiversal package [21] for R.

In the ALLocator workflow (see Figure 1), the first job to execute applies the centWave [5] LC-MS feature detection method of the XCMS [4] software (version 1.26.1) for R. This three-step procedure starts with the creation of m/z-slices, so-called extracted ion base peak chromatograms (EIBPC). Each of these is further processed using a matched filter, which is equivalent to a second derivative Gaussian function. Using the zero crossing points of the resulting filtered chromatogram as integration borders, peaks with a sufficiently high signal-to-noise ratio are integrated in the unfiltered chromatogram. Generated peak tables and the R object are stored to serve as input for the next step in the workflow: spectra deconvolution. Now, two options are available: Either the new ALLocatorSD algorithm for spectra deconvolution, which will be described in detail in the next section, or the CAMERA tool for “compound extraction and annotation” [6], [22]. CAMERA groups peaks based on retention time and peak correlation. Within these peak groups, isotopic peaks are identified and associated with their respective monoisotopic peak. Differences between the m/z-values of all possible pairs of monoisotopic peaks are calculated and matched against a list with differences of common adducts and neutral losses, as well as possible combinations of these.

### The new ALLocatorSD Pipeline for Spectra Deconvolution

**Figure 2:**
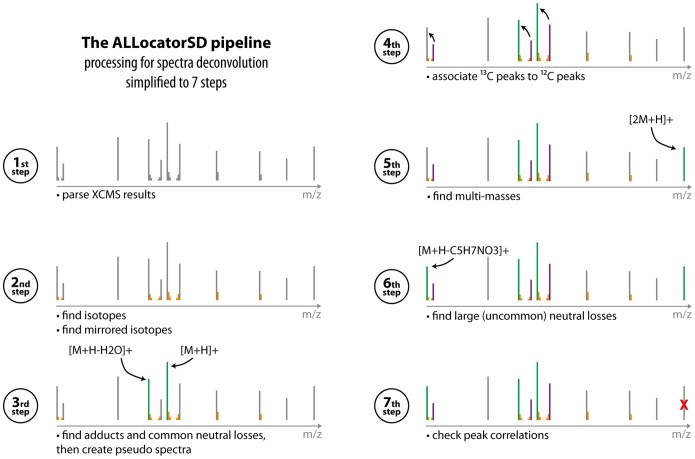
Summary of the ALLocatorSD pipeline for pseudo spectra allocation and peak annotation, divided into seven steps: Reading peaks from the XCMS output (1^st^ step), identification of isotopes (2^nd^ step), computation of pseudo spectra based on common adducts and neutral losses (3^rd^ step), association of ^13^C monoisotopic peaks to ^12^C monoisotopic peaks (4^th^ step), identification of homoadducts (5^th^ step), identification of large (uncommon) neutral losses (6^th^ step), and a check for peak correlations to validate pseudo spectra (7^th^ step).

The paramount purpose of this novel pipeline is to facilitate the interpretation of convoluted mass spectra generated by LC-ESI-MS. This is mainly done by annotating peaks as isotopes, adducts, and fragments, and by associating them to a potential original molecule in a combination of steps (Figure 2). These steps are explained in the following. For the ease of reading, please note that in all steps peaks are only compared to each other, if the deviation in their retention times is less than *ε_rt_*, the allowed retention time error defined by the user. Furthermore, masses and mass-to-charge ratios 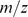 are considered to be equal, if they are within the user-defined accepted mass-to-charge error 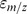. The examples assume a chromatogram that has been acquired in positive mode, but the herein described approach can – without any restriction - also be applied for negative mode measurements.

1^st^ step: To prime spectra deconvolution through the ALLocatorSD pipeline, the list of peaks that have to be annotated (or interpreted) is parsed from the XCMS results mentioned above.

2^nd^ step: Peaks representing isotopologues of another monoisotopic peak have to be identified and associated as such. To this end 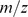 distances of peaks are compared: their masses must increment by 1.003355 Da, while their intensities decrease. Is the distance half of that, this indicates ions that are charged twice. In case of ^13^C-labeling experiments, the same step but for decreasing 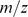 values is repeated to find the lighter isotopologues of monoisotopic ^13^C peaks. These are called *mirrored isotopes* in this manuscript, as isotope patterns of incompletely ^13^C-labeled small molecules resemble a mirrored version of the molecules natural isotopic pattern.

3^rd^ step: In this step, common adducts and neutral losses are searched for. A default list of common adducts and neutral losses is predefined for both, positive and negative acquisition mode, but can easily be changed via the web interface. In these lists, a few adducts (e.g. [M+H]^+^, [M+Na]^+^ and [M+K]^+^) are marked as seed-adducts. The algorithm searches for pairs of monoisotopic ^12^C peaks that have the same 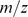 distance as one of the seed-adducts to any other adduct listed (including other seed-adducts). Thus for example, peaks with a distance of 18.0103 Da would be annotated as [M+H-H_2_O]^+^ and [M+H]^+^ of the same mass *M* and another peak with a 21.9819 Da larger weight than the [M+H]^+^ peak would be annotated as the [M+Na]^+^ of *M*. As charges have already been determined (see step 2), even double-charged adducts like [M+2H]^2+^ can be annotated in this step. Step 3 results in a set of pseudo spectra that are generated for a set of masses 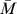. These pseudo spectra consist of peaks that have been annotated as adducts or fragments for each *M* in this step, as well as their isotopologues as detected in step 2.

4^th^ step (applies to ^13^C-labeling experiments only): Identified ^13^C monoisotopic peaks (i.e. they have *mirrored isotopic patterns*) are assigned to their ^12^C counter-parts. A ^13^C peak has to be *n*× 1.003355 Da larger than the ^12^C peak, where *n* is a natural positive number. *n* is further restricted to a range of possible carbon atom occurrences according to mass decomposition. According to this, the associated ^13^C monoisotopic peak of a molecular ion [M+H]^+^ with *n* carbon atoms will be annotated as [M+*n*+H]^+^.

5^th^ step: This step aims to find multi-masses (or homoadducts, i.e. two moieties of the same analyte attached to each other) like [2M+H]^+^, which is obviously easy if *M* is known (see step 3).

6^th^ step: In case of a ^13^C-labeling experiment, step 6 is targeted at finding large (uncommon) neutral losses that have not been predefined (in complement to step 3 which detects predefined neutral losses). A ^12^C peak that is associated to a ^13^C peak with a distance of *n*× 1.003355 Da is expected to have exactly *n* carbon atoms. The algorithm now considers the primary adduct (typically the [M+H]^+^) of any pseudo spectrum, if there exists both a ^12^C and a ^13^C peak, and decomposes its mass with the prerequisite of exactly *n_p_* carbon atoms, resulting in a set of sum formulae *S_p_*. The same is done for each secondary ^12^C/^13^C peak pair with comparatively lower 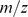 -values and its expected number of carbon atoms *n_f_* that has not been associated to any pseudo spectrum yet, giving *S_f_*. The decomposition of the mass difference between the primary peak pair and the secondary peak pair (i.e. the neutral loss) with a required number of carbon atoms 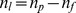 returns *S_l_* as a result. If a unique triplet of sum formulae *s* exists, that explains 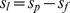, the smaller peak pair can be annotated as a neutral loss [M+H-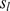]^+^ of the same *M* as the [M+H]^+^.

7^th^ step: In the last step of the procedure, it is checked whether the peaks that were assigned to a pseudo spectrum (all adducts and fragments) correlate well enough: if a peak’s correlation to the primary peak of the pseudo spectrum is worse than a user-defined correlation threshold, it is removed from this pseudo spectrum.

### Differences in the characteristics of ALLocatorSD and CAMERA

With ALLocatorSD and CAMERA two different tools for spectra deconvolution are provided. Both use the XCMS results as input and generate output that can be explored and processed manually using the visualizations and user interface of the ALLocator web platform (see section: data exploration). However, differing output results may be delivered for the same dataset. The most important difference between the two offered methods is the ability of ALLocatorSD to properly process peaks deriving from the addition of U-^13^C-labeled internal standard. Clearly, ALLocatorSD is the recommended deconvolution method for data containing this kind of information.

Besides this major aspect, there are further differences between ALLocatorSD and CAMERA. Firstly, the two tools use different lists of predefined adducts and neutral losses: The number of adducts and neutral losses prearranged in CAMERA is higher, and combinations of these can be detected, too. The respective list in ALLocatorSD is shorter, but customizable through the web interface. Secondly, ALLocatorSD offers an additional level of control by introducing the concept of seed adducts, which have to be present in every pseudo spectrum. For example it can be specified that every pseudo spectrum must contain at least one peak annotated as either the pseudomolecular ion [M+H]^+^ or [M+Na]^+^. Both here described characteristics of ALLocatorSD develop their potential most, if there is some empirical knowledge about the occurrence of certain adducts and neutral losses. The most frequent ion species should be used as seeds, those which are never observed can be excluded from the list.

### Data Exploration and Manual Curation

**Figure 3:**
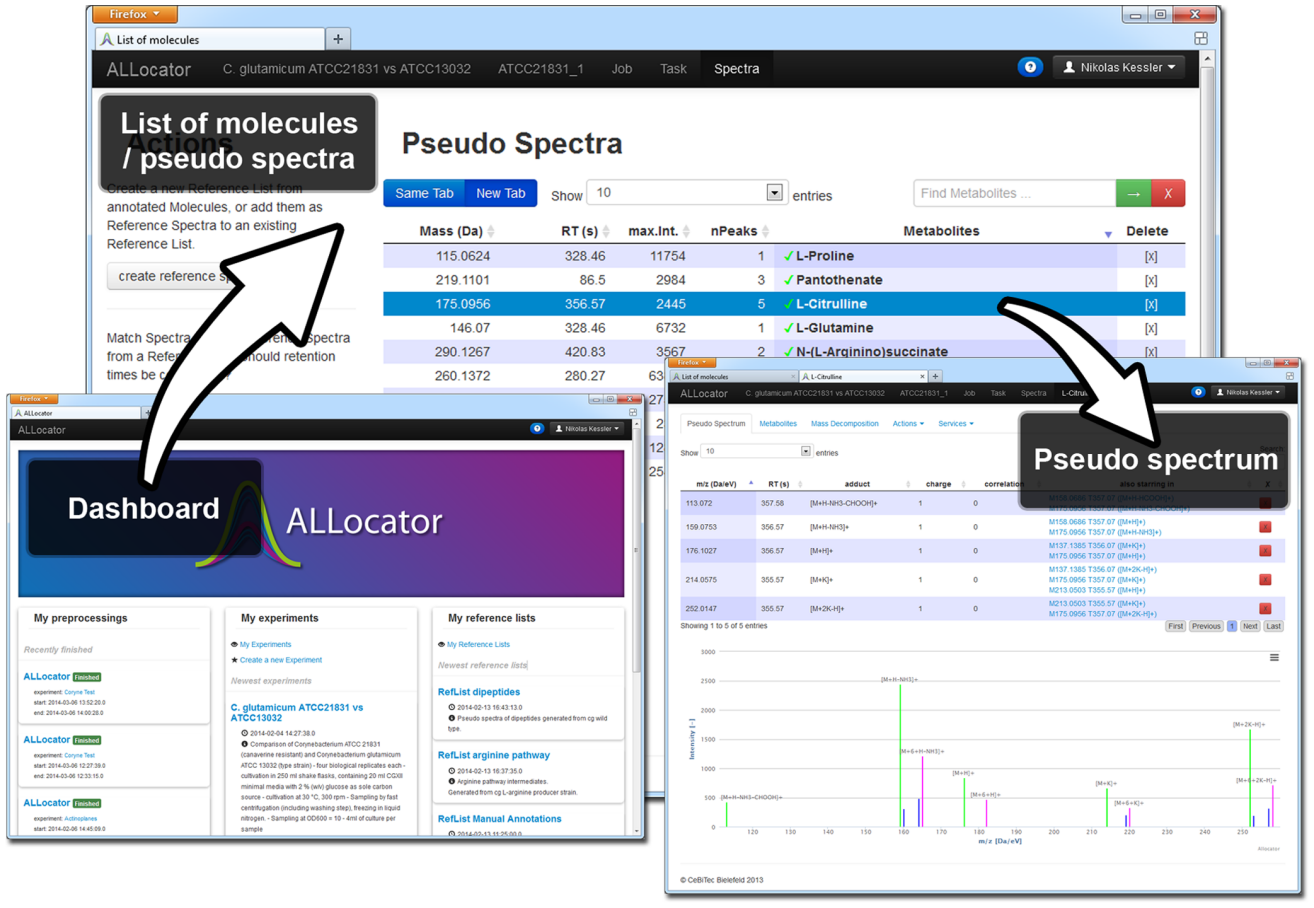
Screenshots from the ALLocator web platform user interface; bottom left: the dashboard that servers as a starting point after log-in; top: The list of molecules or pseudo spectra that were detected in a certain chromatogram; bottom right: A pseudo spectrum view that provides a table of all adducts and fragments, as well as a spectral view of all contributing peaks.

The ALLocator web interface provides several interactive and dynamic views to explore and edit the results generated by the ALLocatorSD processing pipeline (or by CAMERA). In the following bold face names mark tools and views as they are available in the ALLocator user interface, of which some are displayed in Figure 3: The **molecule list view** provides a central table for each chromatogram, which displays all detected pseudo spectra and some relevant information, like the putative mass *M* of the original molecule and a list of KEGG [23], [24] compounds that have the same molecular mass *M*, as well as links to its **pseudo spectrum view**. The **pseudo spectrum view** consists of a table, listing all adducts and losses that were assigned to it, and an interactive pseudo spectrum plot that displays these peaks, their isotopes, and (if available) ^13^C isotopologues. On demand, the extracted ion currents of all the contributing masses can be loaded directly into the view. Using context menus, peaks can be edited or removed. Other correlating peaks can be loaded into the view, and eventually added to the pseudo spectrum. A detailed list of KEGG compounds with the mass *M* is integrated. Additionally, a **spectrum-aware mass decomposition** is integrated, that optionally restricts resulting sum formulae using a variety of intelligent filters (see the section **Spectrum-aware mass decomposition** below). We define ’*spectrum-aware*‘ methods as logics that do not only base on the mass of a pseudo spectrum’s putative molecule, but additionally consider the available fragmentation pattern to generate more precise results.

Aiming to assign a component to each valid pseudo spectrum, an easy to use annotation functionality has been set up. Pseudo spectra can be annotated either by filling a simple **manual annotation** form, or favorably by confirming a *KEGG COMPOUND* with a single click. Hits from the *MassBank* database can be copied into the **manual annotation** form with a single click, too.

Another tool accessible from the **molecule list view** allows browsing ‘orphan peaks’, i.e. peaks that have not been associated to any pseudo spectrum yet. The tool allows filtering these by a retention time window and a minimum intensity. Any orphan peak can be selected as a basis to generate a new pseudo spectrum.

### Integration of Personal and Public Reference Databases

From the **molecule list view** it is possible to create a reference list of all the confirmed or manually annotated compounds, which can then be used in another chromatogram to automatically annotate similar pseudo spectra. The similarity is measured via the dot-product for which a score threshold can be defined. In the **pseudo spectrum view**, the respective single spectrum can be added to (or matched against) a reference list. Additionally, pseudo fragment spectra can be matched against the MassBank MS^2^ database [25] to further interpret the pseudo MS^2^ fragmentation. Pseudo MS^2^ fragmentation can be inferred from many LC-ESI-MS pseudo spectra. Pseudo fragment spectra consist of the pseudo-molecular ion (the [M+H]^+^ or [M–H]^−^) and all its fragments, but exclude any further adducts and ^13^C peaks.

### Spectrum-aware Mass Decomposition

A mass decomposition for the putative mass *M* of any found pseudo spectrum is accessible from the **pseudo spectrum view**. On demand, this view suggests sum formulae that fit to *M*, along with a link to ChemSpider [26]. As the number of theoretical sum formulae increases vastly with the size of *M* and with the accepted mass error *ε_m_*, it is crucial to highly reduce the number of results, without discarding any true positives. Therefore, a set of filters has been implemented. Five of the *Seven Golden Rules* [27] can be activated (filter by element number, element probability, element ratio, Senior rule, Lewis rule), which check sum formulae for chemical plausibility – some of them by chemical rules, others heuristically. We also introduce a new filter that discards all sum formulae with less than 3.3 oxygen atoms per phosphorous atom, as such molecules are rarely (or never) found in the KEGG Compounds database. Additionally, two *spectrum-aware* filters are available: The first considers neutral losses, for example a neutral loss of C_6_H_12_O_6_ in the pseudo spectrum requires all sum formulae to contain at least six carbon, twelve hydrogen and six oxygen atoms. The second new filter that has been implemented considers the ^13^C-labeling information, if available, and can best be explained by an example: if the [M+H]^+^ adduct features a ^13^C peak in distance of 15×1.003355 Da, only sum formulae with fifteen carbon atoms will be presented. As a result, the list of mass decompositions will only contain sum formulae that pass all of the activated filters.

### Data Export for External Use

Data can be exported in several ways and file formats. For each chromatogram, peak lists as well as molecule lists can be exported. Peak lists are basically in the same data format, as generated by the CAMERA functions xsAnnotate and getPeaklist, but extended by one column for the association information of ^12^C and ^13^C isotopologic peaks. Molecule lists contain all confirmed or manually annotated metabolites and the related uniform adducts and fragments. If available, the quotients of the ^12^C and ^13^C ‘abundances’ are given to reflect relative quantities. Molecule lists can be downloaded as a single file for the entire experiment. Here, ‘abundances’ are *intensity*, *area* or *baseline corrected area* as determined by XCMS, divided by the samples biomass or optical density. All files can be downloaded as comma separated files, tab separated files or Microsoft Excel sheets.

### Project Management and Collaboration

All data uploaded to ALLocator is organized into experiments. The creator of the experiment may easily grant and revoke access to other users, but at the same time stays owner of the submitted data. In contrast to typical web services, all raw data uploaded to ALLocator will be stored until single chromatograms or the entire experiment are deleted by one of the authorized users. Downloading of raw data from the platform is not supported. As the web platform is designed in a stateless way, URLs from the web browser address bar can be bookmarked. This can be used for example to inspect a spectrum later or at another working station, as well as to point colleagues towards a specific chromatogram or spectrum. Customized lists of adducts and neutral losses are also protected by permission management and can be shared with other users. In the same way user generated reference spectra can be shared with other users or applied to subsequent experiments. All these features greatly support in-depth analyses of data, distributed collaboration on data, and knowledge transfer between experiments.

## Application Example

### Metabolite Identification in Strains of *Corynebacterium glutamicum* using ALLocator

In this application example the ALLocator web platform was applied for the identification and relative quantitation of abundant metabolites in hydrophilic extracts of the *C. glutamicum* type strain ATCC 13032 and the l-arginine-producing (canavanine resistant) strain *C. glutamicum* ATCC 21831 [28]. Four biological replicates were prepared for both strains and a ^13^C-labeled bacterial extract was used as internal standard. Cultivation of *C. glutamicum* strains, sampling and LC-MS analysis were carried out as described previously by Petri *et al.* [29] and outlined in the experimental section (see Appendix S1). Detailed mass spectrometer settings are listed in Table S1. Experimental raw data and protocols are publicly available (study identifier: MTBLS128) through the MetaboLights repository [30].

All chromatograms were uploaded to ALLocator and organized in a single experiment. Peak detection was performed using XCMS and resulted in the detection of approximately 1,400–1,500 peaks for each chromatogram (for XCMS parameter settings see Table S2). Subsequently, the ALLocatorSD algorithm was started to associate isotopologues and to generate pseudo spectra based on XCMS peak tables (for ALLocatorSD parameter settings see Tables S3 and S4).

The **molecule list view** was then used for manual revision of peak annotations. At first pseudo spectra with a high number of peaks and those containing ^13^C-labeled peaks were reviewed. In addition, substrates and intermediates of the l-arginine biosynthesis pathway were specifically searched for.

**Figure 4:**
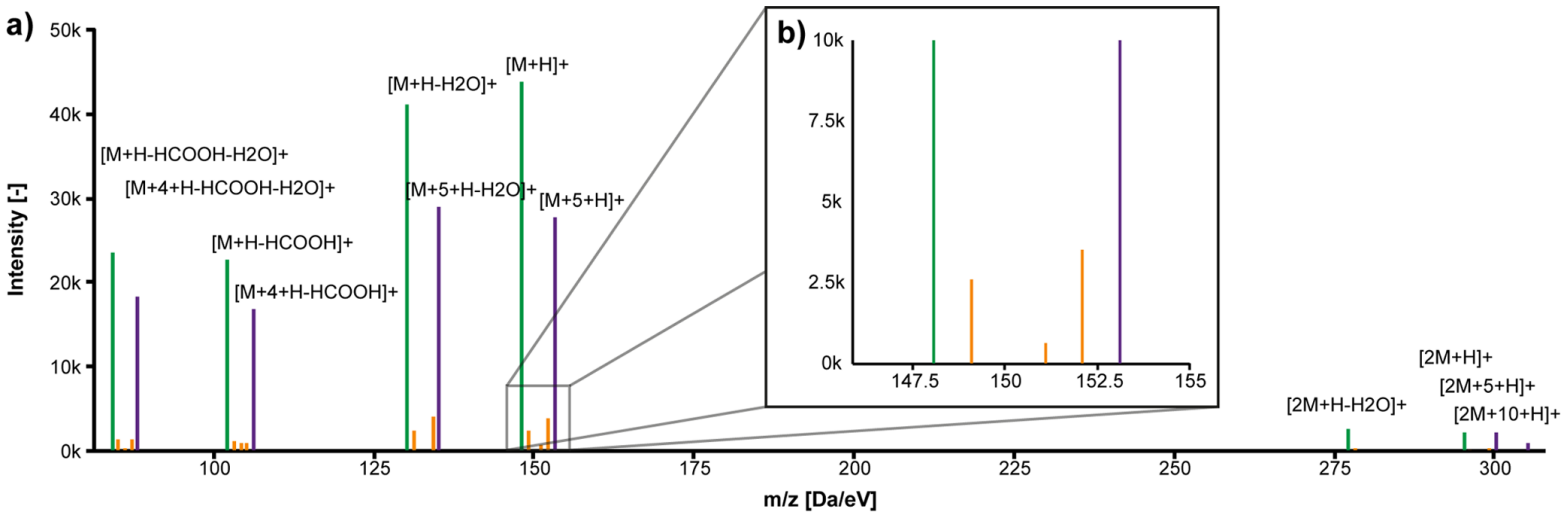
a) Automatically generated pseudo spectrum M147.052T287.92 (glutamic acid); b) zoomed area to depict how ^12^C and ^13^C monoisotopic ions create mirrored isotopic patterns; green: ^12^C monoisotopic peaks; magenta: ^13^C monoisotopic peaks; blue: associated heteroisotopic peaks.

The complete procedure shall be demonstrated by the identification of glutamic acid, the most prominent metabolite and initial substrate for arginine biosynthesis in *C. glutamicum*. Using the search toolbar, the peak list was filtered to solely display metabolites with annotations containing the term “glutamate”. The list included a pseudo spectrum (M147.052T287.92) with six unlabeled peaks of a putative metabolite with a calculated neutral monoisotopic mass of 147.052 Da and a retention time of 288 seconds as depicted in Figure 4. The neutral mass matched 10 entries listed in the KEGG database with a mass deviation of 0.01 Da (see Table S5).

Mass decomposition for 147.052 Da was performed with all available filters activated and finally resulted in only the single formula C_5_H_9_NO_4_. This is indeed the sum formula of glutamate, but also of all other nine metabolites listed in Table S5. A pseudo fragment spectrum was queried against the MassBank database. The best retrieved hit was a spectrum of glutamic acid (Glutamic acid; LC-ESI-QTOF; MS2; CE:15 eV; [M+H]+; MassBank: PB000462) with a score value of above 0.98. In fact, the list of fragment peaks in the pseudo spectrum was identical to that of the MS/MS spectrum of glutamic acid.

All automatically annotated ^13^C-labeled peaks and thereby inferred numbers of carbon atoms were consistent with the annotation of neutral losses, which was initially performed only on the basis of m/z differences. Intensity ratios for all ^12^C monoisotopic peaks to their fully ^13^C-labeled counterparts were similar. One labeled peak (m/z 300.1309) was automatically associated to the [2M+H]^+^ adduct in a distance of +5 Da and annotated as [2M+5+H]^+^. This peak most likely represented an adduct consisting of one unlabeled and one fully ^13^C-labeled isotopoloque. After searching for additional correlating orphan peaks, a peak was identified (m/z 305.1449) representing [2M+10+H]^+^, the adduct of two fully ^13^C-labeled glutamic acid molecules. This peak was manually added to the pseudo spectrum using the **context menu** (see pseudo spectrum in Figure 4a). All available information taken together enabled a reliable identification of glutamate, although no distiction between the l- and d- enantiomer was possible.

A subset of peaks that are associated to a large pseudo spectrum can sometimes be added to an additional pseudo spectrum for another putative mass. This tends to happen when multiple consecutive small neutral losses occur. This shall be demonstrated again using the pseudo spectrum of glutamate (147.0532 Da). Here, three of the peaks were annotated as [M+H-H2O]^+^, [M+H-HCOOH]^+^, and [M+H-HCOOH-H2O]^+^. The same peaks were also assembled to the pseudo spectrum M129.04T287.92 and annotated as [M+H]^+^, [M+H-CO]^+^ and [M+H-HCOOH]^+^, respectively. The putative neutral monoisotopic mass of this second pseudo spectrum (129.04 Da) matched for example 4-oxoproline in the KEGG database. As both pseudo spectra are formally correct when regarded separately and peak correlations can be very good for different coeluting compounds, this ambiguity cannot be solved reliably without manual revision. Thus, it is one of the main goals of the manual editing process to eliminate multiple annotations of such peaks. For this purpose we used the ALLocator function **claim peaks**, which in this case deleted the mentioned peaks from all pseudo spectra except that of glutamate. This is an important advantage over editing annotations in a spread sheet, because it ensures data integrity and the concise visualizations help keeping the overview.

### Annotation of Large Neutral Losses Allows Identification of (γ-)Glutamyl Dipeptides

**Figure 5:**
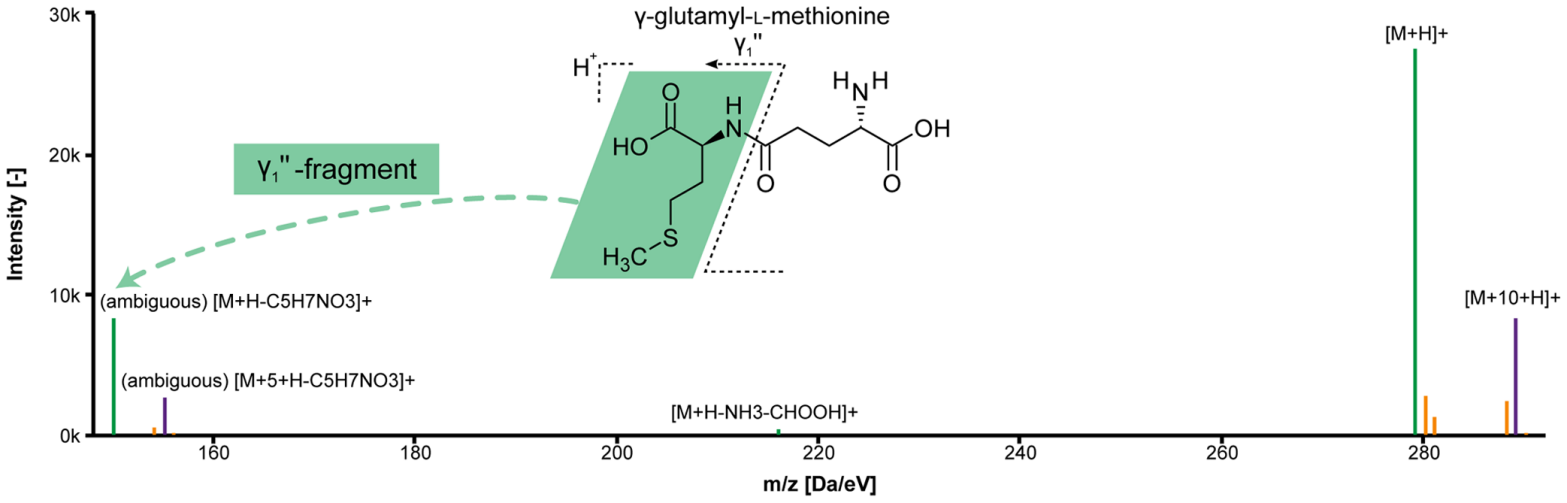
Pseudo spectrum and molecular structure of (γ-)glutamyl-L-methionine. The γ_1_’’-fragment (on the very left) could be annotated thanks to the additional information provided by the ^13^C monoisotopic peaks; green: ^12^C monoisotopic peaks; magenta: ^13^C monoisotopic peaks; blue: associated heteroisotopic peaks; the “(ambiguous)” tag informs the user, that this neutral loss was calculated by mass decomposition and filtered for the correct number of carbon atoms, but still multiple sum formulae might explain the present mass difference.

Amongst the metabolites with the most prominent peaks in both strains we identified several dipeptides. The calculated monoisotopic masses all matched those of at least two different peptides, containing a glutamyl residue at the N- or the C-terminal end. On the basis of the calculated mass alone it was not possible to distinguish between the isobaric compounds, but positional information could be inferred from the generated pseudo spectra. These included peaks for the respective y_1_′′-fragment of the peptide (Figure 5), showing that all dipeptides had an N-terminal glutamyl residue (see Figure 5 and Figures S2, S4-S6). The automatic annotation of the y_1_′′-fragments was possible through the unique ability of ALLocatorSD to deal with ^13^C-labeling experiments. These uncommon fragments are not included in the list of small neutral losses, but could be annotated in the 6^th^ step of the ALLocatorSD pipeline (see Figure 2 and corresponding section). So far we were able to identify the dipeptides as glutamyl-methionine, glutamyl-valine, glutamyl-(iso)leucine and glutamyl-glutamine.

In case of glutamyl-glutamine the y_1_′′-fragment was not assigned by ALLocatorSD. The tool **find correlating peaks** was used with a lowered correlation coefficient threshold of 0.75. The peaks (m/z 147 and m/z 152) representing the expected y_1_′′-fragment and its fully labeled ^13^C isotopologue were present and added to the pseudo spectrum (see Figure S2). Checking the extracted ion chromatograms (EICs, see Figure S3), a different peak shape for these m/z values and a slightly higher retention time compared to the other peaks of the pseudo spectrum was observed. Additionally, the intensity of the fully ^13^C-labeled peak compared to the ^12^C monoisotopic peak was higher than for all the other peak pairs. All these differences could be referred to the coelution of free glutamine, which was checked by the analysis of l-glutamine standard.

Previously, γ-glutamyl-l-glutamine, γ-glutamyl-l-valine, γ-glutamyl-l-leucine and γ-glutamyl-l-glutamate have been isolated from *C. glutamicum* fermentation broths, but the physiological role of these metabolites stayed elusive [31], [32]. Although the presence of [M+H-NH3]^+^ and absence of [M+H-H2O]^+^ ions in the spectra of the before mentioned peptides were an indication for γ-linkages [33], it was not possible to readily distinguish between dipeptides with α- or γ-linkages. This is the first report on the synthesis of (γ-)glutamyl-methionine by *C. glutamicum*, but amongst other γ-glutamyl dipeptides it was detected earlier for example in samples of *Synecococcus* sp. PCC 7002 by an untargeted metabolomics approach [12], [34]. It will be interesting to investigate their functional role in prokaryotic organisms, but further interpretation exceeds the scope of this article.

In order to safe the manual annotation effort and to transfer it to all the other chromatograms in the experiment, the curated pseudo spectra with confirmed metabolite annotations were stored in a reference list using the tool **create reference spectra**. This reference list was later used to automatically detect, assemble and annotate similar pseudo spectra in all the other chromatograms of this experiment using the function **apply reference list**.

### Data Export and Relative Quantitation of Arginine Biosynthesis Intermediates

The identification of bottlenecks by the detection of accumulating pathway intermediates in large libraries of strains is an integral part of modern metabolic engineering strategies and biotechnology [35]. To demonstrate functionalities for export of data and relative quantitation of metabolites, it was obvious to compare the relative abundances of metabolites of the arginine biosynthesis pathway, since *C. glutamicum* ATCC 21831 is an l-arginine producing strain. Here it was possible to identify the substrates l-glutamate and l-glutamine, the intermediates *N*-l-acetylglutamate, l-citrulline and *N*-l-argininosuccinate, as well as the endproduct l-arginine (see Figures S1, S7, S8, S9, S10). For each confirmed metabolite, peak intensities and areas were automatically normalized to internal standard and biomass, and exported to an xls document. Relative quantitation between sample groups and statistics were performed in a spreadsheet (see Table S6). Metabolites mentioned in the following were quantified using the peak areas of the respective [M+H]^+^ ions, and all their abundances were significantly different between strains. The significance was determined by Student’s t-test and multiple testing errors were corrected using the method of Benjamini and Hochberg [36].

The concentration of the initial substrate l-glutamate was lower in the arginine producer than in ATCC 13032 (fold-change 0.23). The intermediate *N*-acetylglutamate was detectable in all samples, but the peaks of the ^13^C-labeled internal standard were below the detection limit, so that no relative quantitation could be performed. As expected, the l-arginine pool was higher (fold-change 12.26) in *C. glutamicum* ATCC 21831 compared to the type strain. But in addition, accumulation of *N*-l-argininosuccinate (fold-change 34.05) and l-citrulline (fold-change 1.9) could be observed, indicating a bottleneck in the last step of arginine production, the conversion of *N*-l-argininosuccinate to arginine and fumarate. This is in good accordance with a recent study by Park *et al.* [37], in which the strain ATCC 21831 (AR0) was used in a systems metabolic engineering approach. Here, authors debottlenecked the last two reactions of the arginine biosynthesis in the derived strain AR6 by replacing the native promoter of the *argGH* operon with a stronger one.

## Conclusion

Correct metabolite identification in LC-ESI-MS datasets heavily relies on expert knowledge and cannot be done automatically *per se*. Due to this, metabolite identification is a major bottleneck in untargeted metabolomics experiments. In addition, stable isotope labeling was reported to greatly facilitate this process.

Introducing ALLocator we provide now a powerful web platform for the semi-automatic annotation of peaks in LC-MS chromatograms and an interface that supports manual improvement of metabolite annotation with interactive tables and visualizations. At the core of this platform we implemented the ALLocatorSD pipeline for the automatic assembly of pseudo spectra. As a major improvement compared to previously existing software, this new algorithm is capable of dealing with ^13^C-labeling experiments, enabling not only relative quantitation (mass isotopomer ratio analysis), but also automatic annotation of fragments resulting from large neutral losses. For the subsequent manual revision and correction of automatic annotation results, the user benefits from the integration of the platform with public metabolite and mass spectral databases (KEGG [23], [24], ChemSpider [26], MassBank [25]) and new powerful tools, as for example the spectrum-aware mass decomposition. The possibility to create, share and query user-defined reference lists is an important feature that ensures transferability of once made annotation efforts to other chromatograms and experiments.

The system contributes to the metabolomics software landscape by extending the bioinformatics coverage of analytical technologies. By supporting LC-ESI-MS data and especially ^13^C SIL it complements the community of metabolomics online platforms, until now constituted by platforms like MeltDB 2.0 [38], XCMSOnline [7], and MetaboAnalyst [39].

In our application example we have demonstrated the applicability of the ALLocator web platform on complex biological samples and used it to annotate and relatively quantify intermediates of the l-arginine biosynthesis in two strains of *C. glutamicum*. Analyzing the data specifically with regard to arginine biosynthesis, the last step of the pathway was identified as a bottleneck in l-arginine production with strain ATCC 21831. In an untargeted manner, we have identified (γ-)glutamyl-methionine as a previously unknown metabolite of *C. glutamicum.* By providing tools for widely automated identification, quantitation and exploration of LC-ESI-MS data, ALLocator is well suited for the processing of LC-ESI-MS datasets in the fields of systems biology and biotechnology.

## Supporting Information

Figure S1 **Pseudo spectrum of l-glutamate.** Green: ^12^C monoisotopic peaks; purple: ^13^C monoisotopic peaks; yellow: associated heteroisotopic peaks.
(TIF)

Figure S2 **Pseudo spectrum of (γ-)glutamyl-glutamine.** Green: ^12^C monoisotopic peaks; purple: ^13^C monoisotopic peaks; yellow: associated heteroisotopic peaks.
(TIF)

Figure S3 **EICs for (γ-)glutamyl-glutamine and l-glutamine.**
(TIF)

Figure S4 **Pseudo spectrum of (γ-)glutamyl-(iso)leucine.** Green: ^12^C monoisotopic peaks; purple: ^13^C monoisotopic peaks; yellow: associated heteroisotopic peaks.
(TIF)

Figure S5 **Pseudo spectrum of (γ-)glutamyl-methionine.** Green: ^12^C monoisotopic peaks; purple: ^13^C monoisotopic peaks; yellow: associated heteroisotopic peaks.
(TIF)

Figure S6 **Pseudo spectrum of (γ-)glutamyl-valine.** Green: ^12^C monoisotopic peaks; purple: ^13^C monoisotopic peaks; yellow: associated heteroisotopic peaks.
(TIF)

Figure S7 **Pseudo spectrum of l-arginine.** Green: ^12^C monoisotopic peaks; purple: ^13^C monoisotopic peaks; yellow: associated heteroisotopic peaks.
(TIF)

Figure S8 **Pseudo spectrum of** ***N*** **-l-argininosuccinate.** Green: ^12^C monoisotopic peaks; purple: ^13^C monoisotopic peaks; yellow: associated heteroisotopic peaks.
(TIF)

Figure S9 **Pseudo spectrum of l-citrulline.** Green: ^12^C monoisotopic peaks; purple: ^13^C monoisotopic peaks; yellow: associated heteroisotopic peaks.
(TIF)

Figure S10 **Pseudo spectrum of** ***N*** **-acetyl-l-glutamate.** Green: ^12^C monoisotopic peaks; yellow: associated heteroisotopic peaks.
(TIF)

Table S1 **Parameters for microTOF control in full scan MS mode.**
(DOC)

Table S2 **Parameters for XCMS as used in the Application Example.**
(DOC)

Table S3 **Parameters for ALLocator as used in the Application Example.**
(DOC)

Table S4 **Adduct list as used in the Application Example.**
(DOC)

Table S5 **Adduct list as used in the Application Example.** Green: ^12^C monoisotopic peaks; purple: ^13^C monoisotopic peaks; yellow: associated heteroisotopic peaks.
(DOC)

Table S6 **Normalized peak areas (int_o) of pseudo-molecular [M+H]^+^ ions of different metabolites.**
(XLS)

Appendix S1 **Experimental section (includes reference** [40] **).**
(DOC)
