# Supplementary figures and images for: ALLocator: An Interactive Web Platform for the Analysis of Metabolomic LC-ESI-MS Datasets, Enabling Semi-Automated, User-Revised Compound Annotation and Mass Isotopomer Ratio Analysis
Source: PLoS One. 2014 Nov 26;9(11):e113909. doi: 10.1371/journal.pone.0113909 (PMC4245236; doi:10.1371/journal.pone.0113909)

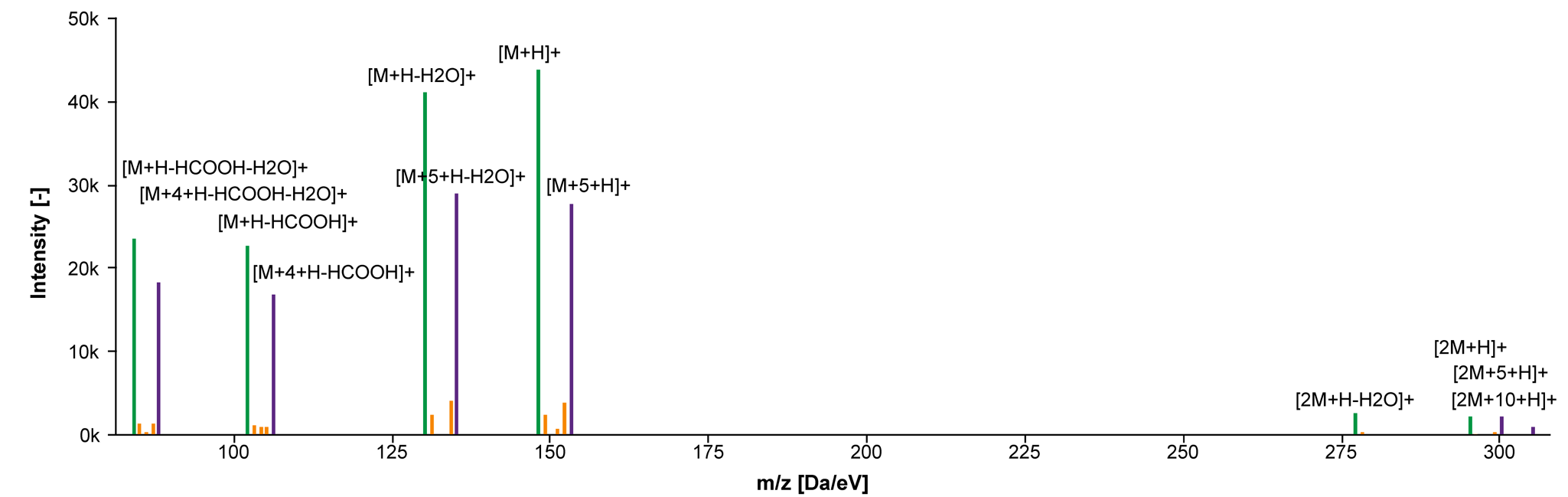

Supplement: Figure S1 — Pseudo spectrum of l-glutamate. Green: 12C monoisotopic peaks; purple: 13C monoisotopic peaks; yellow: associated heteroisotopic peaks. (TIF) [file pone.0113909.s001.tif]

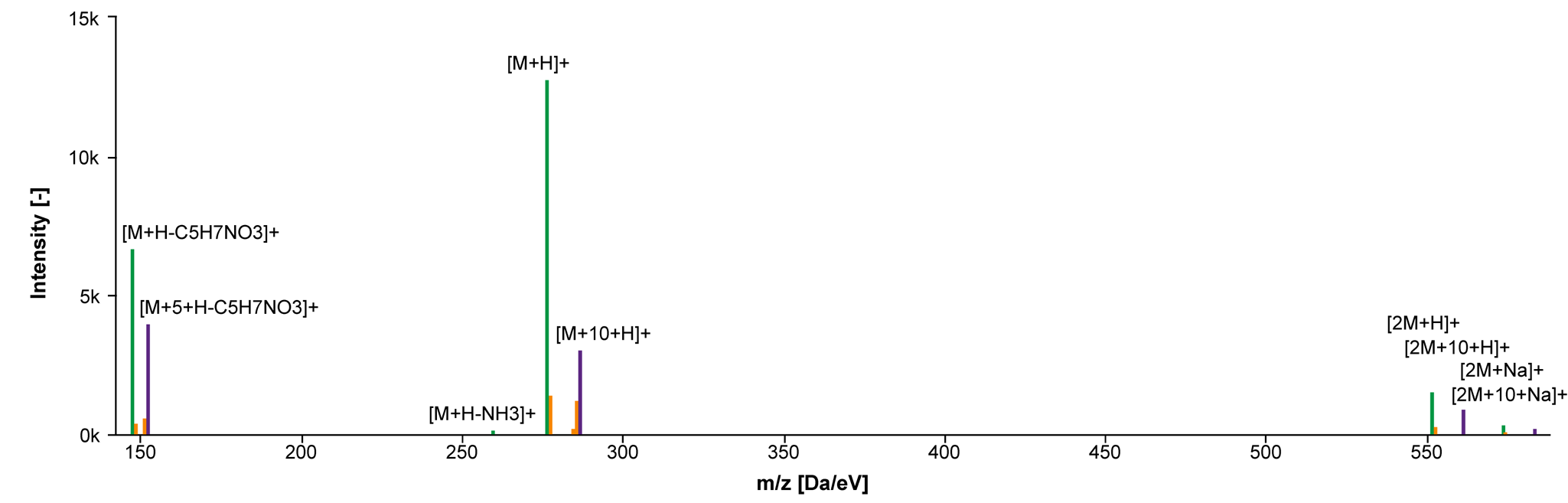

Supplement: Figure S2 — Pseudo spectrum of (γ-)glutamyl-glutamine. Green: 12C monoisotopic peaks; purple: 13C monoisotopic peaks; yellow: associated heteroisotopic peaks. (TIF) [file pone.0113909.s002.tif]

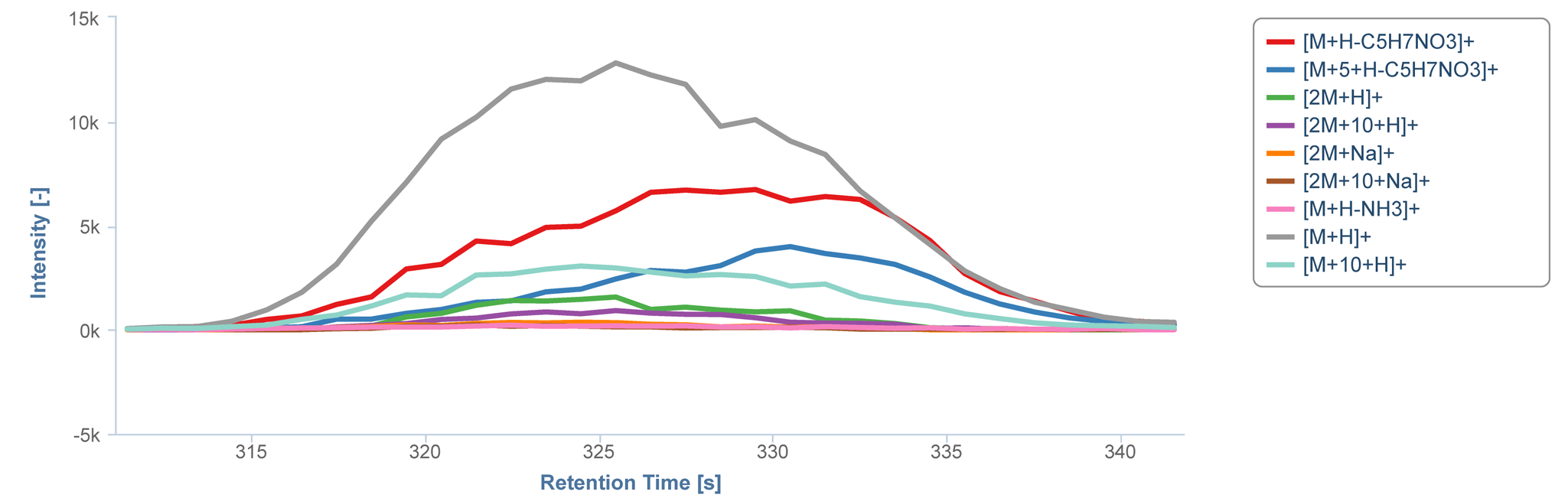

Supplement: Figure S3 — EICs for (γ-)glutamyl-glutamine and l-glutamine. (TIF) [file pone.0113909.s003.tif]

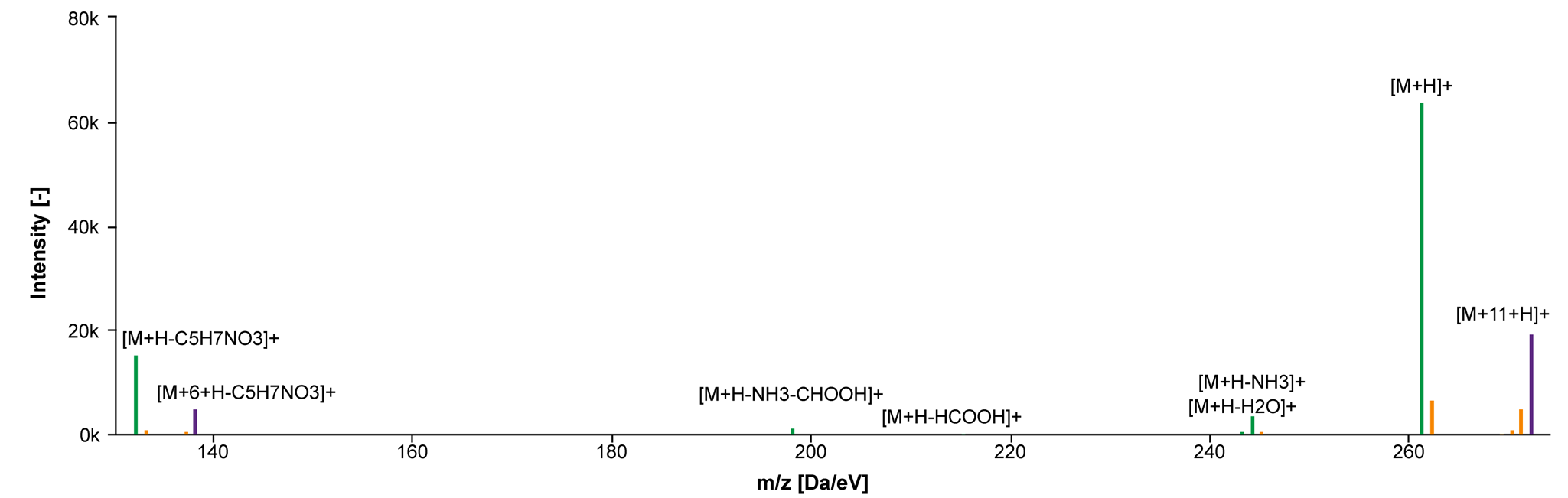

Supplement: Figure S4 — Pseudo spectrum of (γ-)glutamyl-(iso)leucine. Green: 12C monoisotopic peaks; purple: 13C monoisotopic peaks; yellow: associated heteroisotopic peaks. (TIF) [file pone.0113909.s004.tif]

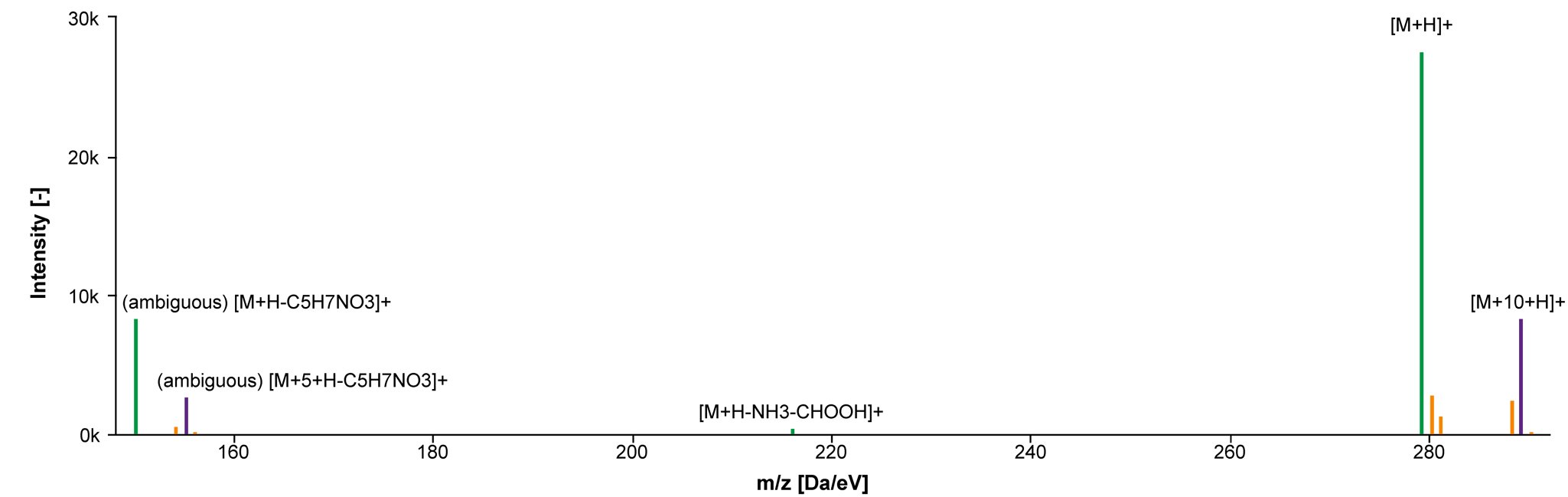

Supplement: Figure S5 — Pseudo spectrum of (γ-)glutamyl-methionine. Green: 12C monoisotopic peaks; purple: 13C monoisotopic peaks; yellow: associated heteroisotopic peaks. (TIF) [file pone.0113909.s005.tif]

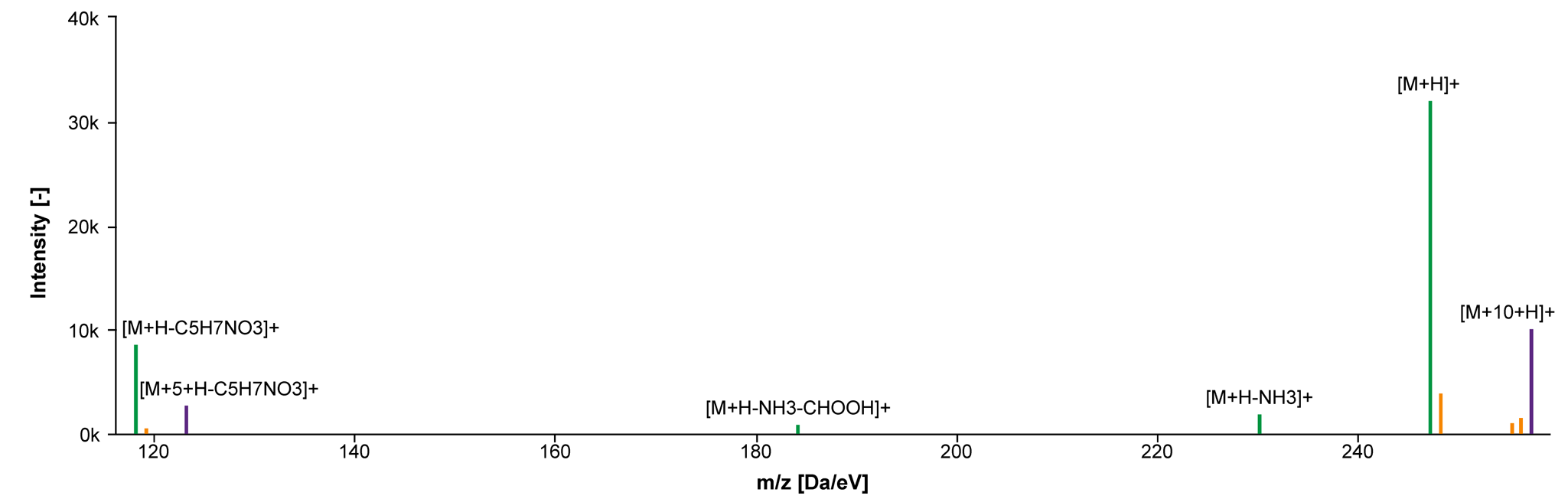

Supplement: Figure S6 — Pseudo spectrum of (γ-)glutamyl-valine. Green: 12C monoisotopic peaks; purple: 13C monoisotopic peaks; yellow: associated heteroisotopic peaks. (TIF) [file pone.0113909.s006.tif]

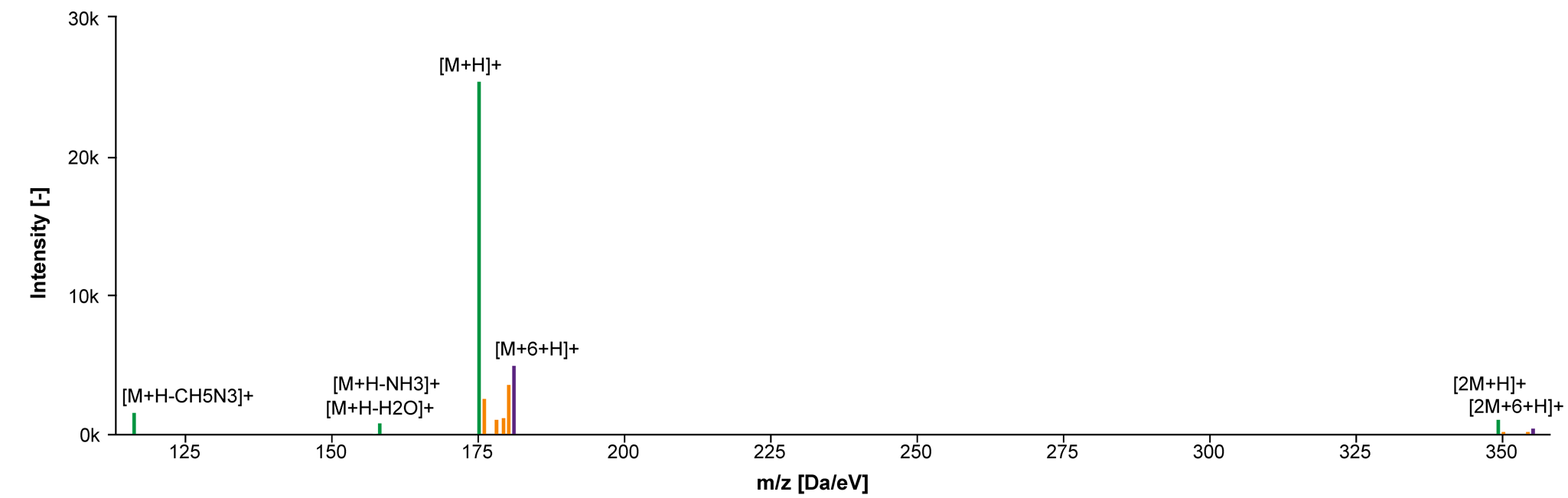

Supplement: Figure S7 — Pseudo spectrum of l-arginine. Green: 12C monoisotopic peaks; purple: 13C monoisotopic peaks; yellow: associated heteroisotopic peaks. (TIF) [file pone.0113909.s007.tif]

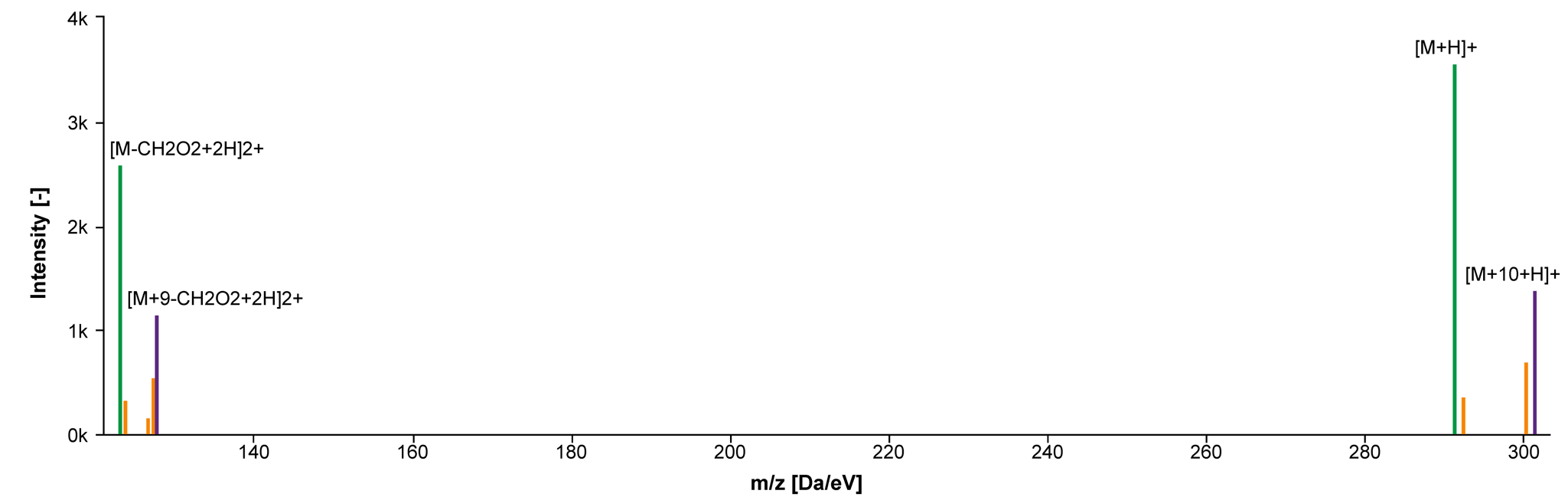

Supplement: Figure S8 — Pseudo spectrum of N -l-argininosuccinate. Green: 12C monoisotopic peaks; purple: 13C monoisotopic peaks; yellow: associated heteroisotopic peaks. (TIF) [file pone.0113909.s008.tif]

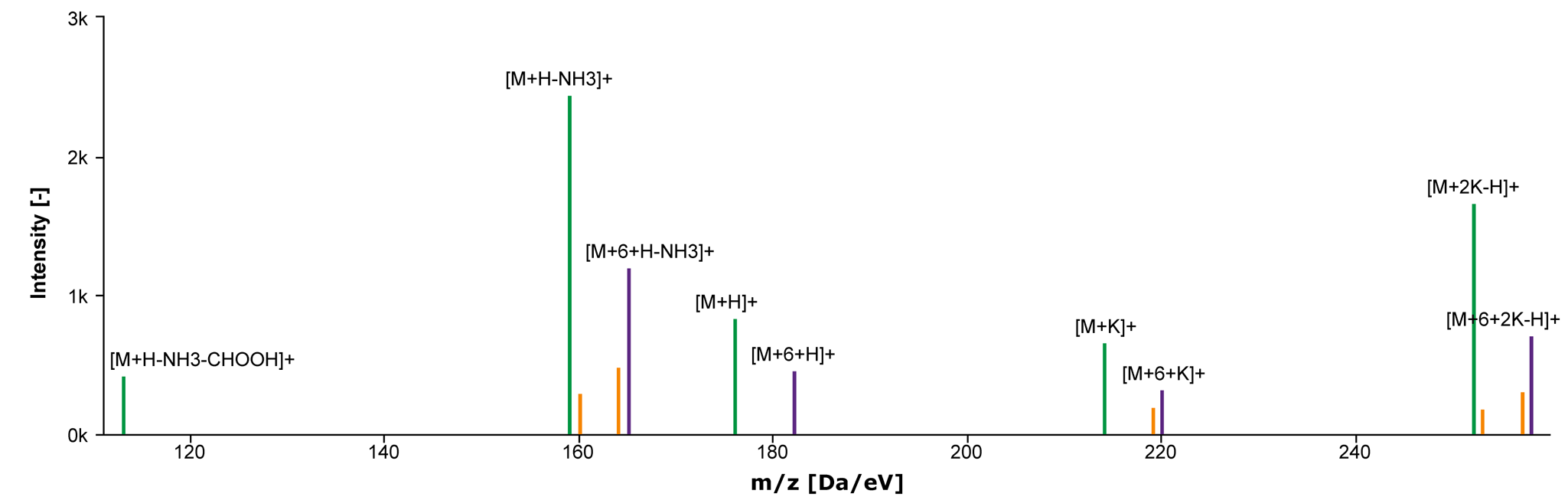

Supplement: Figure S9 — Pseudo spectrum of l-citrulline. Green: 12C monoisotopic peaks; purple: 13C monoisotopic peaks; yellow: associated heteroisotopic peaks. (TIF) [file pone.0113909.s009.tif]

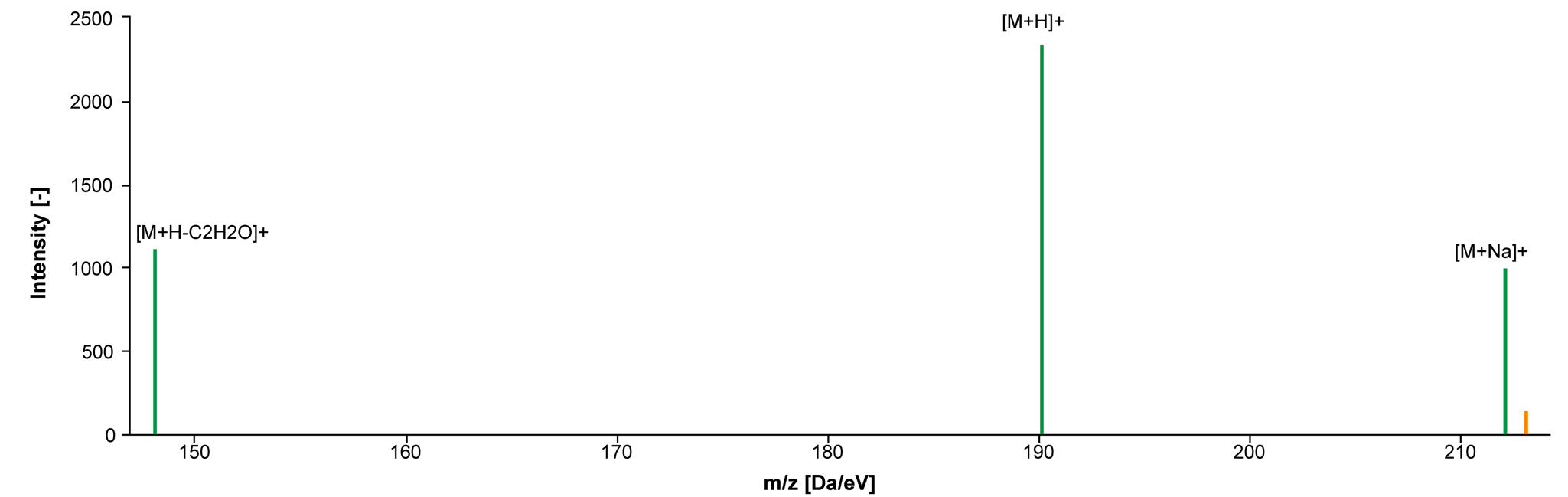

Supplement: Figure S10 — Pseudo spectrum of N -acetyl-l-glutamate. Green: 12C monoisotopic peaks; yellow: associated heteroisotopic peaks. (TIF) [file pone.0113909.s010.tif]
