# Supplementary material for: ALLocator: An Interactive Web Platform for the Analysis of Metabolomic LC-ESI-MS Datasets, Enabling Semi-Automated, User-Revised Compound Annotation and Mass Isotopomer Ratio Analysis
Source: PLoS One. 2014 Nov 26;9(11):e113909. doi: 10.1371/journal.pone.0113909 (PMC4245236; doi:10.1371/journal.pone.0113909)
Supplement: Table S1 — Parameters for microTOF control in full scan MS mode. (DOC) [file pone.0113909.s011.doc]

**Table S1: Parameters for microTOF control in full scan MS mode**

| **Mode** | **Scan Mode** | MS | **Ion Polarity** | Positive |
| --- | --- | --- | --- | --- |
|  | **Mass Range** | 50-1000 m/z | **Rolling Average** | off |
|  | **Spectra Acquisition** | Save Spectra | **Absolute Threshold** | 10 |
|  | **Include Profile Spectra** | Always | **Peak Summation Width** | 5 pts |
|  | **Focus** | Inactive | **Acquisition Rate** | 1.0 Hz |
| **Source** | **Endplate Offset** | -500 V | **Dry Gas** | 8.0 L / min |
|  | **Capillary** | -2500 V | **Dry Temp** | 180 °C |
|  | **Nebulizer** | 3.0 bar |  |  |
| **Transfer** | **Funnel 1 RF** | 180.0 Vpp | **ISCID Energy** | 0.0 eV |
|  | **Funnel 2 RF** | 200.0 Vpp | **Hexapole RF** | 100.0 Vpp |
| **Quadrupole** | **Ion Energy** | 5.0 eV | **Low Mass** | 100.00 m/z |
| **Collision Cell** | **Collision Energy** | 10.0 eV | **Collision RF** | 150.0 Vpp |
|  | **Transfer Time** | 70.0 µs | **Pre Puls Storage** | 7.0 µs |
