# Supplementary material for: ALLocator: An Interactive Web Platform for the Analysis of Metabolomic LC-ESI-MS Datasets, Enabling Semi-Automated, User-Revised Compound Annotation and Mass Isotopomer Ratio Analysis
Source: PLoS One. 2014 Nov 26;9(11):e113909. doi: 10.1371/journal.pone.0113909 (PMC4245236; doi:10.1371/journal.pone.0113909)
Supplement: Table S2 — Parameters for XCMS as used in the Application Example. (DOC) [file pone.0113909.s012.doc]

**Table S2: Parameters for XCMS as used in the Application Example**

| **Parameter** | **Value** |
| --- | --- |
| ppm | 30 |
| peakwidth_min | 10 |
| peakwidth_max | 60 |
| noise | 0 |
| snthresh | 10 |
