# Supplementary material for: ALLocator: An Interactive Web Platform for the Analysis of Metabolomic LC-ESI-MS Datasets, Enabling Semi-Automated, User-Revised Compound Annotation and Mass Isotopomer Ratio Analysis
Source: PLoS One. 2014 Nov 26;9(11):e113909. doi: 10.1371/journal.pone.0113909 (PMC4245236; doi:10.1371/journal.pone.0113909)
Supplement: Table S3 — Parameters for ALLocator as used in the Application Example. (DOC) [file pone.0113909.s013.doc]

**Table S3: Parameters for ALLocator as used in the Application Example**

| **Parameter** | **Value** |
| --- | --- |
| rtBegin | 73 |
| rtEnd | 511 |
| epsilonMZ | 0.005 |
| epsilonRT | 5.0 |
| minCorrelation | 0.75 |
| minIntensity | 0 |
| labeled13C | true |
| omit1000DaMassDecomps | true |
| inputJobId | 86 |
