# Supplementary material for: ALLocator: An Interactive Web Platform for the Analysis of Metabolomic LC-ESI-MS Datasets, Enabling Semi-Automated, User-Revised Compound Annotation and Mass Isotopomer Ratio Analysis
Source: PLoS One. 2014 Nov 26;9(11):e113909. doi: 10.1371/journal.pone.0113909 (PMC4245236; doi:10.1371/journal.pone.0113909)
Supplement: Table S4 — Adduct list as used in the Application Example. (DOC) [file pone.0113909.s014.doc]

**Table S4: Adduct list as used in the Application Example**

| **primary** | **seed** | **common** | **excluded** | **name** |
| --- | --- | --- | --- | --- |
| x |  |  |  | [M+H]+ |
|  |  | x |  | [M+Na]+ |
|  |  | x |  | [M+K]+ |
|  |  | x |  | [M+2Na-H]+ |
|  |  | x |  | [M+2K-H]+ |
|  |  | x |  | [M+H-H2O]+ |
|  |  | x |  | [M+H-H2O-H2O]+ |
|  |  | x |  | [M+H-H2O-H2O-H2O]+ |
|  |  | x |  | [M+H-HCOOH]+ |
|  |  | x |  | [M+H-HCOOH-H2O]+ |
|  |  | x |  | [M+H-C2H4O2]+ |
|  |  | x |  | [M+H-NH3]+ |
|  |  | x |  | [M+H-CO]+ |
|  |  | x |  | [M+H-CO2]+ |
|  |  | x |  | [M+H-C6H10O5]+ |
|  |  | x |  | [M+H-C6H12O6]+ |
|  |  | x |  | [M+H-C6H12O6-H2O]+ |
|  |  | x |  | [M+H-C2H2O]+ |
|  |  | x |  | [M+H-C2H5O2]+ |
|  |  | x |  | [M+H-NH3-CO2]+ |
|  |  | x |  | [M+H-NH3-CHOOH]+ |
|  |  | x |  | [M+H-CH5N3]+ |
|  |  | x |  | [M+H-CH4N2O]+ |
|  |  | x |  | [M+2H]2+ |
|  |  | x |  | [M+2Na]2+ |
