# Supplementary material for: ALLocator: An Interactive Web Platform for the Analysis of Metabolomic LC-ESI-MS Datasets, Enabling Semi-Automated, User-Revised Compound Annotation and Mass Isotopomer Ratio Analysis
Source: PLoS One. 2014 Nov 26;9(11):e113909. doi: 10.1371/journal.pone.0113909 (PMC4245236; doi:10.1371/journal.pone.0113909)
Supplement: Table S5 — Adduct list as used in the Application Example. Green: 12C monoisotopic peaks; purple: 13C monoisotopic peaks; yellow: associated heteroisotopic peaks. (DOC) [file pone.0113909.s015.doc]

**Table S5:** Pseudo spectrum list entry for M147.052T287.92 (glutamic acid) including KEGG Compounds that match the putative mass of 147.052 ± 0.01 Da

| **Mass [Da]** | **Retention Time [s]** | **KEGG Compounds** |
| --- | --- | --- |
| 147.052 | 287.92 | L-Glutamate  2-Oxo-4-hydroxy-5-aminovalerate  L-4-Hydroxyglutamate semialdehyde  Isoglutamate  N-(Carboxymethyl)-D-alanine  L-threo-3-Methylaspartate  O-Acetyl-L-serine  DL-Glutamate  D-Glutamate  N-Methyl-D-aspartic acid |
