# Supplementary material for: ALLocator: An Interactive Web Platform for the Analysis of Metabolomic LC-ESI-MS Datasets, Enabling Semi-Automated, User-Revised Compound Annotation and Mass Isotopomer Ratio Analysis
Source: PLoS One. 2014 Nov 26;9(11):e113909. doi: 10.1371/journal.pone.0113909 (PMC4245236; doi:10.1371/journal.pone.0113909)
Supplement: Appendix S1 — Experimental section (includes reference [40] ). (DOC) [file pone.0113909.s017.doc]

**Appendix S-1: Experimental section**

**Bacterial Strains and Growth Conditions**

*C. glutamicum* strains ATCC 13032 and ATCC 21831 were grown on CASO solid agar plates at 30 °C for 36 h. They were inoculated in 10 mL of CGXII medium (pH 7), consisting of (per liter) 40 g 3‑morpholinopropanesulfonic acid, 20 g (NH_4_)_2_SO_4_, 5 g urea, 1 g KH_2_PO_4_, 1 g K_2_HPO_4_, 0.25 g MgSO_4_ ∙ 7H_2_O, 10 mg FeSO_4_ ∙ 7H_2_0, 10 mg MnSO_4_∙ 7H_2_0, 1 mg ZnSO_4_∙ 7H_2_0, 0.2 mg CuSO_4_, 0.02 mg NiCl_2_ ∙ 6H_2_0, 0.2 mg biotin, 0.42 mg thiamine, 0.03 mg protocatechuate and 20 g glucose [36], transferred into 100 mL shake flasks and incubated o/n at 30 °C and 300 rpm. Main cultures, carried out in 250 mL shake flasks with 20 mL CGXII medium, were inoculated from these precultures to an initial optical density of OD_600_ = 0.4. Cultures were incubated at 30 °C and 300 rpm until mid-exponential growth phase (OD_600_ = 10). Cell harvesting, metabolite extraction and sample preparation were performed as described previously [28]. For each strain, four biological replicates were prepared. For the generation of ^13^C-labeled internal standard, *C. glutamicum* ATCC 21831 was grown in shake flasks with [U^13^C]-glucose (1 % w/v, Cambridge Isotope Laboratories) as sole carbon source.

**Highperformance Liquid Chromatography (HPLC)/ ESI-Quadrupole Time-of-flight (QTOF)MS Analysis**

LC/MS data were obtained using a LaChromUltra (Hitachi Europe, UK) HPLC system coupled to a MicroTOF‑Q-II hybrid quadrupole time‑of‑flight mass spectrometer (Bruker Daltonik, Bremen, Germany), equipped with an electrospray ionization (ESI) source. Chromatographic separation of metabolites was performed on a Cogent diamond hydride column (MicroSolv Technologies; 150 x 2.1 mm; 3 µm particles) operated in aqueous normal phase mode. Eluent A: 50 % acetonitrile (v/v), 50 % water (v/v) + 0.1 % (v/v) formic acid and B: 90 % acetonitrile (v/v), 10 % water (v/v) + 0.1 % (v/v) formic acid were prepared freshly and stored in PTFE bottles. Flow rate was set to 400 µL min^-1^ and linear gradient elution was performed as follows: *t* = 0 min, 100 % B; *t* = 6 min 0 % B; *t* = 7 min, 0 % B; *t* = 8.5 min 100 % B; *t* = 13 min 100 % B. MS detection was performed with ESI source operated in positive ionization mode (see Table S-1). Nitrogen was used as sheath, dry, and collision gas. Sodium formate solution (0.1 M) in 50 % (v/v) isopropanol was used for external mass calibration and was injected into the ESI source at the beginning of each analysis, using a switch valve and a segmented acquisition method. Spectra were obtained with a scan rate of 1 Hz and within an m/z range of 50 - 1000.

**Recalibration of LC/MS data and file conversion**

Automatic internal mass calibration using an HPC quadratic algorithm and file conversion to netCDF-file format was achieved using Compass DataAnalysis™ version 4.0 SP1 (Bruker Daltonik, Bremen, Germany).
